# Rectus abdominis muscle atrophy, prophylactic mesh, and stoma placement: retrospective findings from a prospective multicenter trial

**DOI:** 10.1007/s10029-025-03309-8

**Published:** 2025-04-28

**Authors:** Staffan Täckström, Eva Angenete, Rode Grönkvist, Eva Haglind, Peter Kälebo, Adiela Correa Marinez, Jacob Rosenberg, Maziar Nikberg

**Affiliations:** 1 Department of Radiology, Västmanlands Hospital Västerås, Västerås, Sweden; 2 Department of Surgery, Västmanlands Hospital Västerås, Västerås, Sweden; 3 Centre for Clinical Research of Uppsala University, Västerås, Sweden; 4 Department of Surgery, Scandinavian Surgical Outcomes Research Group, Institute of Clinical Sciences, Sahlgrenska Academy, University of Gothenburg, Gothenburg, Sweden; 5 Department of Surgery, Region Västra Götaland, Sahlgrenska University Hospital/Östra, Gothenburg, Sweden; 6 Department of Surgery, Herlev and Gentofte Hospital, University of Copenhagen, Herlev, Denmark; 7 Department of Radiology, Sahlgrenska University Hospital, Gothenburg, Sweden; 8 School of Public Health and Community Medicine, Institute of Medicine, Sahlgrenska Academy, University of Gothenburg, Gothenburg, Sweden

**Keywords:** Parastomal hernia, Prophylactic mesh, Rectus abdominis muscle atrophy, Risk factors

## Abstract

**Purpose:**

The primary aim of this study was to investigate whether rectus abdominis muscle atrophy is associated with a lower risk of developing parastomal hernia. Secondary objectives were to assess whether the use of prophylactic mesh is a risk factor for rectus abdominis muscle atrophy and whether the position of the stoma within the rectus abdominis muscle affects the risk of parastomal hernia.

**Methods:**

This retrospective study analysed patients from a prospective, randomised, multicentre trial in which rectal cancer patients were randomised to stoma creation with or without prophylactic mesh. Computed tomography at 12 months was evaluated to identify parastomal hernia, rectus abdominis muscle atrophy and position of stoma in the rectus abdominis muscle.

**Results:**

Out of 149 patients, rectus abdominis muscle atrophy was observed in 9% and parastomal hernia in 42% of patients. There was no association between rectus abdominis muscle atrophy and parastomal hernia (*p* = 0.80; RR 1.07; CI 0.62–1.86), nor between prophylactic mesh and rectus abdominis muscle atrophy (*p* = 0.19; RR 2.00; CI 0.7–5.73). Stoma placement within the rectus abdominis muscle also showed no association with parastomal hernia development (*p* = 0.69; RR 0.97; CI 0.81–1.15).

**Conclusion:**

This study found no statistically significant association between rectus abdominis muscle atrophy and parastomal hernia. The use of prophylactic mesh was not a risk factor for rectus abdominis muscle atrophy, and stoma placement within the rectus abdominis muscle was not associated with parastomal hernia. The previously reported association between prophylactic mesh, rectus abdominis muscle atrophy, and parastomal hernia was not confirmed in this cohort.

**Supplementary Information:**

The online version contains supplementary material available at 10.1007/s10029-025-03309-8.

## Background

Parastomal hernia is a common and challenging complication following stoma surgery. It may affect quality of life [1], as well as cause problems with bandaging [2] and thus require surgery [3]. The incidence of parastomal hernia has been reported as high as 53% at 12 months follow-up evaluated using computed tomography (CT) [4]. The precise causes of parastomal hernia are largely unknown, although some risk factors such as the size of the stomal aperture [5] and high body mass index (BMI) [6, 7] have been reported. However, the results after repair for parastomal hernia are often disappointing and due to high recurrence of parastomal hernia after parastomal hernia repair, focus has moved to prevention of parastomal hernia [6, 8–11].

The role of prophylactic mesh in preventing parastomal hernia is controversial. While some studies reported beneficial outcomes [8, 9], others found no significant benefit [6, 10, 11]. Prophylactic mesh placement is recommended by the European Hernia Society [12, 13], but this has not been widely adopted [14] due to the conflicting results mentioned above and probably also due to the extended operating time and costs associated with prophylactic mesh.

A retrospective study from our research group identified the use of prophylactic mesh as a potential risk factor for rectus abdominis muscle atrophy [15], which could potentially be attributed to the injury of intercostal nerves during the dissection for prophylactic mesh placement. Surprisingly, rectus abdominis muscle atrophy was identified as a protective factor for developing parastomal hernia in that study.

The primary aim of this study was to investigate our previous findings, within a cohort of patients from a prospective randomized multicenter trial, if rectus abdominis muscle atrophy was associated with a lower risk of developing parastomal hernia one year after stoma construction. Secondary objectives were to examine whether the use of prophylactic mesh constituted a risk factor for the development of rectus abdominis muscle atrophy and if the placement of the stoma within the rectus abdominis muscle was associated with an increased risk of parastomal hernia.

## Methods

### Study cohort

The Stoma-Const trial randomized patients into one of three groups: one with prophylactic mesh, and two without prophylactic mesh with the stoma created by circular or cruciate incision. Patients [6] were recruited from two hospitals in Sweden and one in Denmark; however, only the two Swedish hospitals randomized also to a mesh group. The original inclusion criteria for Stoma-Const were patients scheduled for elective end colostomy, who had no previous abdominal hernia, and who consented to participate in the study. Patients were recruited between June 2013 and September 2017. Due to the specific research question addressed in this retrospective substudy, only patients included in the two hospitals in Sweden that randomized patients to a mesh group were included. Further, in this analysis the prophylactic mesh group was compared to the combined group cruciate and circular incision, i.e. no prophylactic mesh.

### Surgical procedures

Details on the surgical technique are described in the original Stoma-Const article [6]. The prophylactic meshes were 10 × 10 cm lightweight and partially absorbable (Ultrapro, ETHICON, Johnson & Johnson) placed in a sublay position, dorsal to the rectus abdominis muscle and anterior to the posterior rectus fascia and anchored to the posterior rectus sheath with absorbable sutures 2 − 0. The choice between laparoscopic and open method was at the discretion of the surgeon.

### Hernia diagnosis

All patients were scheduled for a follow-up with abdominal computed tomography after 12 months, both in a supine position and, for many patients, a series in a prone position with a ring around the stoma for optimal assessability regarding parastomal hernia which was defined as either herniation of intra-abdominal content through the abdominal wall or the demonstration of a hernia sac. When CT was absent, the parastomal hernia diagnosis was based on clinical assessment. The assessment was made in the original Stoma-Const trial by one experienced radiologist who was blinded regarding surgical technique (PK). The prophylactic mesh was not visible on computed tomography. In this study patients without a follow-up CT were excluded.

### Atrophy of the rectus abdominis muscle

The thickness of the rectus abdominis muscle beneath the stoma was assessed in comparison to the contralateral side by two experienced radiologists (ST and PK) blinded to the surgical technique used. If it was visually deemed thinner it was classified as atrophied; otherwise, it was deemed equal. A consensus was reached following discussion between the two radiologists in cases where their evaluations differed. In these instances, both radiologists reviewed the images together, with the radiologist who had identified muscle atrophy demonstrating the finding to the other, followed by a discussion until consensus was reached.

### Risk factors

**Fig. 1:**
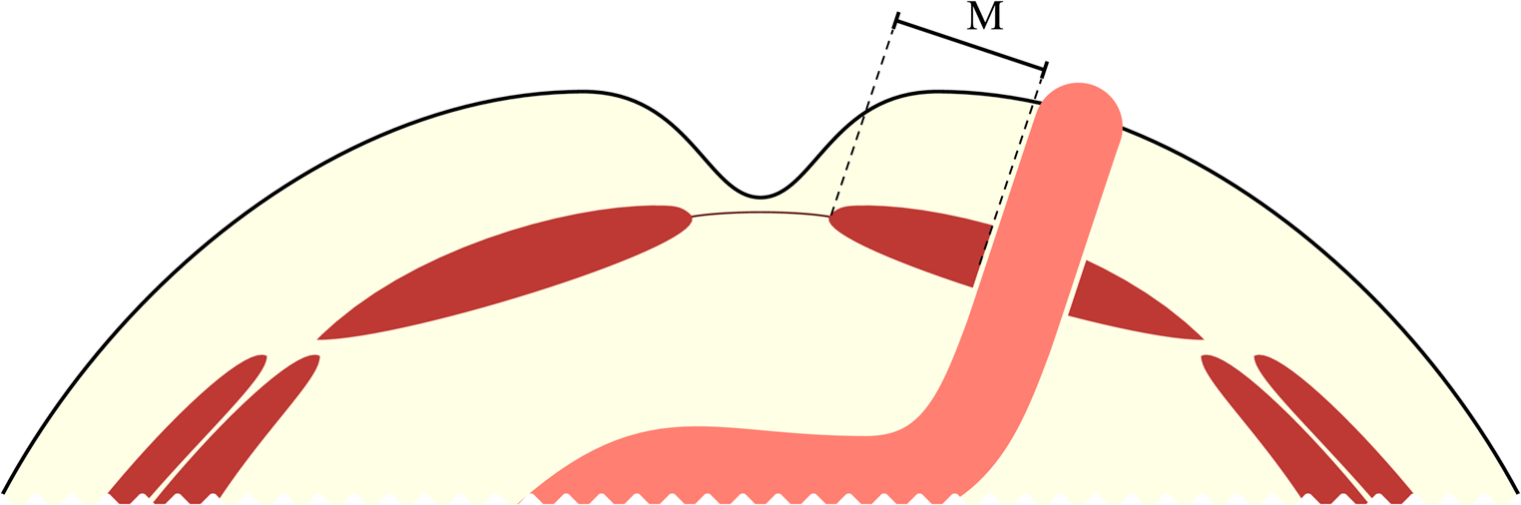
Measurement of the position of the stoma in the rectus abdominis muscle in mm, between the medial edge of the muscle and the medial edge of the stoma

The position of the stoma in the rectus abdominis muscle was measured on computed tomography by a radiologist at the 12-month follow-up. The distance was measured in a computed tomography slice on the axial slice corresponding to the center of the stoma, from the medial edge of the rectus abdominis muscle to the medial edge of the stoma (Fig. 1).

The diameter of the stoma was measured during surgery and is described in more detail elsewhere [6]. The diameter was used to calculate the stoma area by approximating it as a circle. Age, BMI, sex, comorbidity, and surgical method (open or laparoscopic) were prospectively recorded. Comorbidity in the Stoma-Const trial was defined as the patient having a history of at least one of the following comorbidities: stroke, thromboembolic disease, hypertension, diabetes, cardiovascular disease, or lung disease.

### Statistical analysis

A statistical analysis plan was created before any statistical analyses were conducted (supplementary material). Based on the estimated incidence of rectus abdominis muscle atrophy from a previous study [15], the study was powered to detect a difference in proportion of 30% with 80% power for the primary endpoint. For the secondary endpoint, which investigated the risk ratio of surgical techniques for developing rectus abdominis muscle atrophy, assuming the same incidence, the statistical power was greater than 99.9%. The analyses were conducted according to a per-protocol principle. Missing values were handled by listwise deletion. For both primary and secondary endpoints the modified Poisson regression model [16] was used, controlling for covariates. The results are presented as risk ratios (RR), 95% confidence intervals, and adjusted P-values. *P* < 0.05 was considered statistically significant.

Interrater agreement for the two radiologists’ assessment of rectus abdominis muscle atrophy was calculated using Cohen’s kappa.

All statistical analyses were performed with the R Statistical software [17].

## Results

**Fig. 2:**
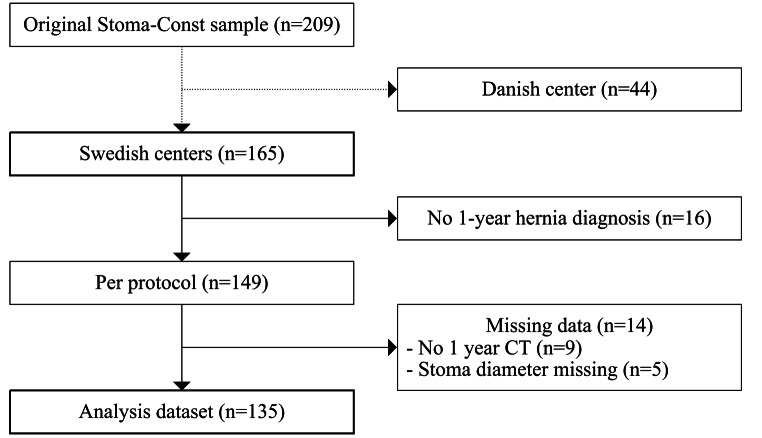
Flowchart of patient inclusion, exclusion and analysis cohort

Of the 209 patients included in the original Stoma-Const trial, 165 were included in the two Swedish centers and therefore included in the present study. Of these, 16 were excluded because there was no clinical or radiological follow-up at 12 months, resulting in 149 patients per protocol. Computed tomography was absent in 9 additional patients and therefore excluded. A total of 14 patients had missing data, resulting in 135 patients remaining for analysis (Fig. 2).

Descriptive data of these patients are shown in Table 1. The mean age was 68 years and 56% of patients were male. At the one-year follow-up, rectus abdominis muscle atrophy was observed in 13 patients (9%) and parastomal hernia in 63 patients (42%).

**Table 1:** Clinical and radiological data on patients with a colostomy with and without a prophylactic stoma mesh

	No mesh (*N* = 92)	Mesh (*N* = 57)	Overall (*N* = 149)
Sex			
Female	47 (51.1%)	19 (33.3%)	66 (44.3%)
Male	45 (48.9%)	38 (66.7%)	83 (55.7%)
BMI			
Mean (SD)	26 (4.6)	27 (4.9)	26 (4.7)
Age (years)			
Mean (SD)	68 (12)	67 (11)	68 (12)
Comorbidity			
No	45 (48.9%)	32 (56.1%)	77 (51.7%)
Yes	47 (51.1%)	25 (43.9%)	72 (48.3%)
RAM atrophy			
No	82 (89.1%)	45 (78.9%)	127 (85.2%)
Yes	6 (6.5%)	7 (12.3%)	13 (8.7%)
Missing	4 (4.3%)	5 (8.8%)	9 (6.0%)
Parastomal hernia at 1 year			
No	51 (55.4%)	35 (61.4%)	86 (57.7%)
Yes	41 (44.6%)	22 (38.6%)	63 (42.3%)
Surgical method			
Laparoscopic	32 (34.8%)	19 (33.3%)	51 (34.2%)
Open	60 (65.2%)	38 (66.7%)	98 (65.8%)
Stoma area (cm^2)			
Mean (SD)	9.7 (3.2)	11 (3.4)	10 (3.3)
Missing	4 (4.3%)	2 (3.5%)	6 (4.0%)
Stoma position (medial stoma border to medial RAM limit, cm)			
Mean (SD)	3.3 (1.3)	3.3 (1.1)	3.3 (1.2)
Missing	3 (3.3%)	5 (8.8%)	8 (5.4%)

SD standard deviation, RAM rectus abdominis muscle, BMI body mass index

### Parastomal hernia and rectus abdominis muscle atrophy

There was no association between rectus abdominis muscle atrophy and parastomal hernia one year after surgery (*p* = 0.80; RR 1.07; CI 0.62–1.86) (Table 2). A significant effect on parastomal hernia was found for age (*p* = 0.01; RR 1.03 CI 1.01–1.03) and a borderline significance for area of the stoma (*p* = 0.05; RR 0.93; CI 0.87-1.00).

**Table 2:** Modified Poisson regression of PSH risk in groups with and without RAM atrophy one year after surgery

	RR	95% CI	*p*
RAM atrophy			0.80
No	1.0		
Yes	1.07	0.62–1.86	
Age	1.03	1.01–1.05	**0.01**
BMI	1.04	0.99–1.09	0.15
Sex			0.77
Female	1.00		
Male	0.94	0.63–1.41	0.77
Surgical approach			0.55
Laparoscopic	1.0		
Open	1.15	0.73–1.80	
Stoma aperture area (cm^2^)	0.93	0.87-1.00	0.05
Stomal position in the RAM (cm)	0.97	0.81–1.15	0.70
Comorbidity			0.89
No	1.0		
Yes	1.03	0.67–1.59	

P-values < 0.05 are in bold

RR relative risk, CI confidence interval, RAM rectus abdominis muscle, BMI body mass index, PSH parastomal hernia

No association between prophylactic mesh and rectus abdominis muscle atrophy was found (*P* = 0.19; RR 2.00, CI 0.7–5.73) (Table 3). The interrater agreement of the radiologists regarding rectus abdominis muscle atrophy was high (Cohen’s kappa 0.85).

**Table 3:** Risk ratio between patients with and without prophylactic mesh for RAM atrophy

	RR	95% CI	*p*
Prophylactic mesh			0.19
No mesh	1.00		
Mesh	2.00	0.70–5.73	
Age	1.00	0.95–1.06	0.97
BMI	0.99	0.86 − 1.13	0.86
Sex			0.68
Female	1.0		
Male	1.23	0.45–3.35	
Surgical approach			0.11
Laparoscopic	1.0		
Open	3.19	0.78–13.06	
Stoma aperture area (cm^2^)	0.81	0.68–0.98	**0.03**
Stomal position in the RAM (cm)	0.94	0.51–1.71	0.83
Comorbidity			0.99
No	1.0		
Yes	0.99	0.26–3.76	

P-values < 0.05 are in bold

RR relative risk, CI confidence interval, RAM rectus abdominis muscle, BMI body mass index

### Position of stoma in rectus abdominis muscle

There was no association between stoma placement in rectus abdominis muscle, measured from its medial limit, and developing parastomal hernia (*p* = 0.69; RR 0.97; CI 0.81–1.15) (Table 4).

**Table 4:** Risk ratio of different stoma placement for PSH

	RR	95% CI	*p*
Age	1.03	1.01–1.05	**0.01**
BMI	1.04	0.99–1.09	0.14
Sex			0.77
Female	1.0		
Male	0.94	0.63–1.40	
Surgical approach			0.53
Laparoscopic	1.0		
Open	1.15	0.74–1.80	
Stoma aperture area (cm^2^)	0.93	0.87–1.00	0.04
Stomal position in the RAM (cm)	0.97	0.81–1.15	0.69
Comorbidity	1.03		0.89
No	1.0		
Yes	1.03	0.67–1.59	

P-values < 0.05 are in bold

RR relative risk, CI confidence interval, RAM rectus abdominis muscle, BMI body mass index, PSH parastomal hernia

## Discussion

In this study, rectus abdominis muscle atrophy was not common with only 9% of patients identified, but 42% of patients had parastomal hernia one year after surgery. Rectus abdominis muscle atrophy was not identified as a significant protecting factor for parastomal hernia and prophylactic mesh placement was not a significant risk factor for rectus abdominis muscle atrophy.

The finding that rectus abdominis muscle atrophy was not a significant factor for parastomal hernia differs from the results of a previous study. A notable distinction from that study [15] was the considerably lower incidence of rectus abdominis muscle atrophy observed in patients receiving a prophylactic stoma mesh which reduced the study’s power to detect a significant effect. The reasons behind this discrepancy remain unclear. Similar stoma construction techniques (sublay) and mesh size (10 × 10 cm) were employed in both studies. Moreover, one of the radiological reviewers in this study also served as the sole reviewer in the previous study, suggesting that the interpretation of rectus abdominis muscle atrophy should not differ markedly. The high interrater agreement also indicates that this assessment can be considered relatively valid.

We did not find that rectus abdominis muscle atrophy was a risk factor for the development of a parastomal hernia. A retrospective study [7] yielded contrasting results, wherein rectus abdominis muscle atrophy was identified as a risk factor for parastomal hernia. The differences between that study and ours are numerous: the definition of parastomal hernia (clinical vs. computed tomography), a significantly lower incidence of parastomal hernia (24% vs. 42%), and the absence of prophylactic mesh but most important the design difference: retrospective single center study versus prospective, randomized multicenter trial. In contrast to our earlier study, rectus abdominis muscle atrophy was assessed using measurements at multiple points (four points each on the left and right rectus abdominis muscle, laterally and medially, both cranially and caudally to the stoma) [7]. Therefore, comparing the two studies becomes challenging. The complexity of identifying risk factors for parastomal hernia and the eventual relationship between rectus abdominis muscle atrophy and parastomal hernia may also be influenced by surgical practices and patient characteristics, but the surgical method was not detailed in the previous study [7].

A possible explanation for the occurrence of rectus abdominis muscle atrophy during stoma mesh placement is the inadvertent dissection at the level of the neurovascular bundle, which could potentially damage the 11th and 12th intercostal and the iliohypogastric nerves, innervating the rectus abdominis muscle below the stoma. More extensive dissection possibly occurs during open surgery compared to laparoscopic techniques. In the earlier study [15], all surgeries were performed using an open approach, whereas in this study, 66% were. This may partly explain the lower incidence of rectus abdominis muscle atrophy but could not account for the entire difference. It remains plausible that individual surgeons have differing dissection techniques and perhaps this could be responsible for the difference in the prevalence of rectus abdominis muscle atrophy. In Stoma-Const the protocol included a clear description including a video of dissection techniques to be used during creation of the stoma, possibly leading to less inadvertent damage to structures around the stoma, which could in part explain the different outcomes in the two trials.

There is no validated method to assess rectus abdominis muscle atrophy using computed tomography in the context of patients with a stoma. We considered two different approaches: measuring a specific metric, such as thickness or area, at a certain position relative to the stoma site or visually assessing whether the rectus abdominis muscle was thinner than on the contralateral side. The first option might seem more objective and reproducible, but during the creation of a stoma, the muscle is divided at an arbitrary location, and a large stoma can also widen and stretch the muscle making it thinner even if atrophy has not occurred. To measure muscle thickness at a fixed point, defined by anatomical structures, could potentially be misleading as the thickness could be due to thinning/atrophy of the muscle or due to altered shape. Instead, we defined atrophy as a definite difference in the muscle thickness compared to the contralateral side, observed visually.

Previous studies have indicated a positive relationship between the area of the stoma site and parastomal hernia. However, these studies had a methodological issue as the stoma site was measured on the postoperative computed tomography where parastomal hernia was assessed, but it is conceivable that a large parastomal hernia could expand the stoma site, thereby complicating the evaluation of causality. In Stoma-Const, the stoma diameter was measured perioperatively, thus eliminating this issue. Therefore, the finding in this study that a larger stoma area was not associated with a significant increase (*p* = 0.05; RR = 0.93; CI 0.87-1.00) in the risk for parastomal hernia contradicts the associations previously suggested.

When comparing results from different parastomal hernia studies, three main challenges become apparent. First, the methods used to diagnose parastomal hernia vary, with some studies relying on computed tomography scans and others on clinical assessments. Second, there is considerable variation in the reported cases of parastomal hernia, which is partly due to differences in the diagnostic criteria used in computed tomography imaging and clinical evaluations. Third, the studies have been conducted using a range of surgical techniques, including open and laparoscopic approaches, as well as various methods for stoma creation and mesh placement.

### Strengths and limitations

This was a retrospective analysis of a prospective, randomized, multicenter trial, focusing on surgical techniques and the use of prophylactic mesh. As such, many of the strengths inherent in prospective trials are retained. The effects of rectus abdominis muscle atrophy on parastomal hernia can only be interpreted as an association.

A notable strength of the study lies in the strictly defined surgical technique and the uniform mesh size used throughout the study. However, this also limits its generalizability, as variations in surgical techniques and mesh sizes may differently affect the abdominal musculature and adjacent nerves.

Additionally, the lower than anticipated rate of rectus abdominis muscle atrophy may have resulted in a type II error in the analysis of its relationship with parastomal hernia risk, as indicated by the wide confidence intervals observed in the Poisson regression analysis.

Furthermore, approximately 10% of the patients were excluded because they did not undergo follow-up computed tomography imaging at the one-year mark. Nevertheless, we believe the likelihood of any systematic bias affecting our data interpretation is low.

## Conclusions

No significant difference was observed in the parastomal hernia incidence between patients with rectus abdominis muscle atrophy and those without. Additionally, the use of prophylactic mesh did not constitute a risk factor for the development of rectus abdominis muscle atrophy in this study. The previously reported association between prophylactic mesh placement, rectus abdominis muscle atrophy and parastomal hernia could not be verified in this cohort.

## Electronic supplementary material

Below is the link to the electronic supplementary material.

Supplementary Material 1

Supplementary Material 2

## Data Availability

Not applicable.
