# Supplementary material for: Rectus abdominis muscle atrophy, prophylactic mesh, and stoma placement: retrospective findings from a prospective multicenter trial
Source: Hernia. 2025 Apr 28;29(1):151. doi: 10.1007/s10029-025-03309-8 (PMC12037428; doi:10.1007/s10029-025-03309-8)
Supplement: Supplementary file 2 — Supplementary Material 2 [file 10029_2025_3309_MOESM2_ESM.docx]

**Statistical Analysis Plan**

*Rectus abdominis muscle atrophy - a risk factor for parastomal hernia? – a report from Stoma-Const, a randomized controlled trial*

VERSION 2.0

Table of contents

[INTRODUCTION 3](#_Toc147764273)

[ANALYSIS OBJECTIVES 3](#_Toc147764274)

[STUDY METHODS 3](#_Toc147764275)

[ANALYSIS POPULATION, TYPE OF ANALYSIS 3](#_Toc147764276)

[Analysis population 3](#_Toc147764277)

[Type of analysis 3](#_Toc147764278)

[VARIABLES AND ENDPOINTS 4](#_Toc147764279)

[Secondary objectives 4](#_Toc147764280)

[Covariates, stratum and subgroups 4](#_Toc147764281)

[HANDLING OF MISSING VALUES AND OTHER DATA CONVENTIONS 5](#_Toc147764282)

[STATISTICAL METHODOLOGY 5](#_Toc147764283)

[General principles 5](#_Toc147764284)

[Multiplicity 5](#_Toc147764285)

[Interpretation of results 5](#_Toc147764286)

[Type of analyses 5](#_Toc147764287)

[Sample size 6](#_Toc147764288)

[Demography and patient characteristics 6](#_Toc147764289)

[Primary endpoint 6](#_Toc147764290)

[Secondary endpoints 7](#_Toc147764291)

[ADDITIONAL ANALYSES 7](#_Toc147764292)

[TECHNICAL DETAILS 7](#_Toc147764293)

[LIST OF TABLES AND FIGURES PLANNED FOR THE MANUSCRIPT 7](#_Toc147764294)

[DIRECT ACYCLIC GRAPH 8](#_Toc147764295)

[Dagitty code 8](#_Toc147764296)

[REFERENCES 8](#_Toc147764297)

[APPENDIX 9](#_Toc147764298)

[Table 1 Variables and endpoints 9](#_Toc147764299)

# INTRODUCTION

This Statistical Analysis Plan (SAP) details the statistical and data related aspect of a planned manuscript on assessing whether rectus abominis muscle atrophy (*defined as a thinner muscle on the stoma side as seen on a postoperative computer tomography (CT) scan*) constitutes a protective factor for parastomal hernia (*defined as any herniation of intraabdominal content beyond the abdominal wall or a hernia sac identified in the prone or supine position on CT scan (if only the latter was available)*) as well as whether surgical technique used in colostomy construction constitutes a risk factor for rectus abdominis muscle atrophy and whether stoma placement (*measured from the medial limit of the muscle on 1-year CT scan)* constitutes a risk factor for parastomal hernia.

# ANALYSIS OBJECTIVES

The primary objective is to investigate if rectus abdominis muscle atrophy is a risk factor for development of parastomal hernia one year after surgery.

Secondary objectives are:

1. Are any of the investigated operative techniques risk factors for developing rectus abdominis muscle atrophy?
2. Is stoma placement measured from the medial limit of the rectus abdominis muscle a risk factor for developing parastomal hernia?

# STUDY METHODS

Data are drawn from the Stoma-Const study[[1](#_ENREF_1)], a prospective randomized study comparing the difference in risk for developing parastomal hernia one year after surgery with patients randomized to one of three surgical techniques:

- Cruciate incision (control)
- Circular incision
- Reinforcement of abdominal wall with a mesh around the stoma

The data material from StomaConst was deemed appropriate for use in a repeat retrospective analysis of rectus abdominis muscle atrophy and stoma placement in the rectus abdominis muscle.

# ANALYSIS POPULATION, TYPE OF ANALYSIS

## Analysis population

The analysis population consists of all patients who were included in the original study for whom a one-year CT examination was performed at all Swedish centres.

## Type of analysis

The patients will be analyzed per protocol.

VARIABLES AND ENDPOINTS
Primary objective

The primary endpoint is the risk ratio between groups with and without rectus abdominis muscle atrophy for having parastomal hernia one year after surgery. The primary endpoint is derived from a modified Poisson regression model controlling for several covariates.

The variables associated with the primary objective are presence of rectus abdominis muscle atrophy <*varname_RAMatr>* and parastomal hernia at one year <*varname_Parastomal hernia>*.

Presence of rectus abdominis muscle atrophy will be determined by simultaneous retrospective examination by two radiologists of one-year CT examinations. Both assessors will examine the same image material, note their own judgment (rectus abdominis muscle atrophy yes/no) and then reach a consensus judgment. Cohen’s kappa will be reported as a measure of inter-rater agreement.

The determination of parastomal hernia at one year performed in the Stoma-Const trial will also be used in this study.

Further details are given in Table 1.

Secondary objectives

The secondary endpoints are the risk ratios between surgical techniques for developing RAM atrophy, and the risk ratio of a difference of stoma placement in RAM, measured from its medial limit, for developing parastomal hernia, respectively. The secondary endpoints are derived from modified Poisson regression models controlling for several covariates.

For the surgical techniques endpoint, the alternative technique of reinforcement of abdominal wall with a mesh around the stoma will be compared to the control techniques of cruciate incision and circular incision.

The variables associated with the secondary objectives are surgical technique <*varname_surgtec>* and presence of RAM atrophy <*varname_RAMatr>*, and stoma placement in RAM <*varname_stomaloc>* and parastomal hernia at one year <*varname_* parastomal hernia*>*, respectively.

Stoma placement in RAM will be determined by one radiologist assessor, measuring the distance from the medial RAM limit to the medial stoma border in mm.

Further details are given in Table 1.

## Covariates, stratum and subgroups

Models for primary and secondary endpoints will be adjusted for potentially influential background variables by including as covariates the following variables:

1. Age
2. BMI
3. Sex
4. Method of surgery (laparoscopic/open surgery)
5. Comorbidity as defined in the Stoma-Const trial including diabetes and cardiovascular disease. Defined as yes or no to one of several co-morbidity questions (question 15 in preoperative questionnaire)
6. Stoma aperture area (from the perioperative CRF question 16, fascia incision diameter
7. Stoma placement in the rectus abdominis muscle (where it is not the endpoint variable) measured by one radiologist as the distance from the midline to the beginning (medial) of the stoma aperture

These covariates will be investigated for potential multicollinearity before modelling. If serious multicollinearity is detected, some variables may be excluded.

Further details are given in Table 1.

# HANDLING OF MISSING VALUES AND OTHER DATA CONVENTIONS

Missing values will be handled by listwise deletion in all analyses.

# STATISTICAL METHODOLOGY

## General principles

### Multiplicity

In accordance with study objectives, several comparisons will be made. To test the primary endpoint one risk ratio will be tested for significant difference from 1. To test the secondary endpoints, one comparison will be made for the endpoint concerning the effect of surgical technique on rectua abdominis muscle atrophy (mesh vs cruciate or circular incision) and one comparison will be made for the endpoint concerning the effect of stoma location on the parastomal hernia rate. We are aware of the risk for a type 2 error, but have chosen to perform the analyses with this knowledge and will interpret results with causion.

### Interpretation of results

Due to randomization of surgical technique in the data material, any estimated effects of surgical technique on risk of rectus abdominis muscle atrophy can be interpreted causally.

Estimated effects of rectus abdominis muscle atrophy on risk of parastomal hernia and of stoma location on risk of rectus abdominis muscle atrophy cannot be interpreted causally. Instead, any such effects should be interpreted as correlational.

### Type of analyses

The primary analyses for all endpoints will be adjusted analyses. Existence of serious multicollinearity as well as potential interaction effects between the explanatory variable and/or the covariates will be explored.

## Sample size

The StomaConst study based its sample size on a power computation using a two-sided proportion test using normal approximation of the binomial distribution to obtain 80% power for a significance level of 0.05 and a true difference in proportion of 20%. This gave a sample size of 62 per group (three groups, one for each surgical technique).

For the present study, only patients for whom a one-year CT examination was performed at one of the Swedish centers are included, giving a sample size of 140. Approximating the incidence of rectus abdominis muscle atrophy as 37% for the mesh arm and 2% for the cruciate/circular arm (following Täckström 2022)[[2](#_ENREF_2)], 21 subjects are expected to display rectus abdominis muscle atrophy. Using the same test and significance level as above and assuming 44% incidence of parastomal hernia in the group without rectus abdominis muscle atrophy (following Correa Marinez 2020)[[1](#_ENREF_1)], this allows detection of a difference in proportion of 30% with 80% power for the primary endpoint.

For the secondary endpoint investigating risk ratio of surgical techniques for developing rectus abdominis muscle atrophy, the surgical technique arms consist of 88 subjects (cruciate/circular) and 52 subjects (mesh) respectively. Assuming rectus abdominis muscle atrophy incidence per arm as above, this gives >99.9% power.

Power for the secondary endpoint investigating risk ratio of stoma position for developing parastomal hernia is not easily computable in advance without knowledge of the distribution of stoma position in the sample. Thus, this power computation will be performed post-hoc.

## Demography and patient characteristics

*<Example:*

- *Age (median (Q1 ; Q3))*
- *BMI (median (Q1 ; Q3))*
- *Gender (frequency(%))*
- *tumour stage (frequency(%)) >*

Pending dataset

## Primary endpoint

The primary endpoint will be investigated using modified Poisson regression models using a sandwich estimator to compute confidence intervals and p-values, following Zou (2004), with parastomal hernia at one year as the response and MRA as a covariate. Other covariates to be adjusted for are stated in the “Covariates” section of the SAP. If clear overdispersion is detected in response data, it will instead be modelled using a negative binomial model.

The surgical technique variable will be included as a covariate in the model, as it is a potential confounder.

The result will be presented as a risk ratio for parastomal hernia at one year between MRA yes/no groups with 95% confidence interval and p-value for testing the null hypothesis of no difference in risk.

## Secondary endpoints

The secondary endpoints will be investigated using modified Poisson regression models using a sandwich estimator to compute confidence intervals and p-values[[3](#_ENREF_3)]. For the secondary endpoints concerning surgical techniques, MRA at one year will be included as the response and surgical technique as covariates. For the secondary endpoint concerning stoma placement, parastomal hernia at one year will be included as the response and stoma placement will be included as a covariate. Other covariates to be adjusted for are stated in the “Covariates” section of the SAP. If clear overdispersion is detected in response data, it will instead be modelled using a negative binomial model.

For the secondary endpoint concerning stoma placement, the surgical technique variable will be included as a covariate in the model, as it is a potential confounder.

The results for the endpoints concerning surgical technique will be presented as risk ratios for MRA between surgical technique groups with 95% confidence intervals and p-values for testing the null hypotheses of no difference in risk.

The results for the endpoints concerning stoma placement will be presented as risk ratios for parastomal hernia at one year for a unit difference in stoma placement with 95% confidence interval and p-value for testing the null hypothesis of no difference in risk.

# ADDITIONAL ANALYSES

None planned

# TECHNICAL DETAILS

Software used are:

- R®.

# LIST OF TABLES AND FIGURES PLANNED FOR THE MANUSCRIPT

Figure 1: Flow chart of included patients

Table 1: Patient demographics (overview of StomaConst, also the subset included in this study)

Table 2: Frequency rectus abdominis muscle atrophy yes/no, stoma position vs surgical technique (columns surgical technique, rows rectus abdominis muscle atrophy and stoma position)

Table 3: Results for primary and secondary endpoints

Table 4: Analysis of risk factors (other model covariates)

# DIRECT ACYCLIC GRAPH

www.dagitty.net

### Dagitty code

dag {

bb="-4.843,-4.178,3.772,4.524"

"Mesh/No mesh" [pos="-0.125,-3.452"]

"Open/laparoscopy" [pos="-2.478,-2.181"]

"Rectus abdominis muscle atrophy" [exposure,pos="0.586,-0.093"]

"Stoma aperture" [pos="-4.125,0.439"]

"Stoma placement in the rectus abdominis muscle" [pos="-1.144,2.404"]

"parastomal hernia" [outcome,pos="-0.605,-0.502"]

Age [pos="2.074,-2.559"]

BMI [pos="1.634,2.119"]

Comorbidity [pos="3.054,0.088"]

Sex [pos="0.853,3.799"]

"Mesh/No mesh" -> "Rectus abdominis muscle atrophy"

"Mesh/No mesh" -> "parastomal hernia"

"Open/laparoscopy" -> "Rectus abdominis muscle atrophy"

"Open/laparoscopy" -> "parastomal hernia"

"Rectus abdominis muscle atrophy" -> "parastomal hernia"

"Stoma aperture" -> "parastomal hernia"

"Stoma placement in the rectus abdominis muscle" -> "Rectus abdominis muscle atrophy"

"Stoma placement in the rectus abdominis muscle" -> "parastomal hernia"

Age -> "Rectus abdominis muscle atrophy"

Age -> "parastomal hernia"

BMI -> "Stoma placement in the rectus abdominis muscle"

BMI -> "parastomal hernia"

BMI -> Comorbidity

Comorbidity -> "Rectus abdominis muscle atrophy"

Comorbidity -> "parastomal hernia"

Sex -> "Rectus abdominis muscle atrophy"

}

# REFERENCES

1. Correa Marinez, A., et al., *Methods of Colostomy Construction: No Effect on Parastomal Hernia Rate: Results from Stoma-const-A Randomized Controlled Trial.* Ann Surg, 2021. **273**(4): p. 640-647.

2. Täckström, S., et al., *Use of prophylactic stoma mesh is a risk factor for developing rectus abdominis muscle atrophy.* Hernia, 2022.

3. Zou, G., *A modified poisson regression approach to prospective studies with binary data.* Am J Epidemiol, 2004. **159**(7): p. 702-6.

# APPENDIX

## Table 1 Variables and endpoints

*<Analysis Database Specific Derivation Template.xls>*
